# Correction: Factors influencing surgecon implementation in four Canadian emergency departments guided by consolidated framework for implementation research

**DOI:** 10.1371/journal.pone.0343785

**Published:** 2026-02-25

**Authors:** The PLOS One Staff

**Notice of Republication**

This article was republished on February 12, 2026, to correct errors in the title and references that were introduced during the typesetting process. The publisher apologizes for the errors. Please download this article again to view the correct version. The originally published, uncorrected article and the republished, corrected articles are provided here for reference.

## Supporting information

S1 File Originally published, uncorrected article. (PDF)

S2 File Republished, corrected article. (PDF)
