# Supplementary material for: Correction: Factors influencing surgecon implementation in four Canadian emergency departments guided by consolidated framework for implementation research
Source: PLoS One. 2026 Feb 25;21(2):e0343785. doi: 10.1371/journal.pone.0343785 (PMC12935210; doi:10.1371/journal.pone.0343785)
Supplement: S2 File — (PDF) [file pone.0343785.s002.pdf]

PLOS One

RESEARCH ARTICLE

# Factors Influencing SurgeCon Implementation in Four Canadian Emergency Departments Guided by Consolidated Framework for Implementation Research

**Nahid Rahimipour Anaraki[1], Meghraj Mukhopadhyay[1], Christopher Patey[2,3], Paul Norman[2], Jennifer Jewer[4], Holly Etchegary[3], Oliver Hurley[1], Anna Walsh[1], Dorothy Mary Senior[1], Peizhong Peter Wang[3], Shabnam Asghari[1,3]***

**1** Centre for Rural Health Studies, Faculty of Medicine, Memorial University of Newfoundland, St. John's, Canada, **2** Eastern Health, Carbonear Institute for Rural Reach and Innovation by the Sea, Carbonear General Hospital, Carbonear, Canada, **3** Faculty of Medicine, Memorial University of Newfoundland, St. John's, Canada, **4** Faculty of Business Administration, Memorial University of Newfoundland, St. John's, Canada

* sasghari@mun.ca

OPEN ACCESS

**Citation:** Rahimipour Anaraki N, Mukhopadhyay M, Patey C, Norman P, Jewer J, Etchegary H, et al. (2025) Factors Influencing SurgeCon Implementation in Four Canadian Emergency Departments Guided by Consolidated Framework for Implementation Research. PLoS One 20(12): e0337389. https://doi.org/10.1371/journal.pone.0337389

**Editor:** Saima Aleem, Khyber Medical University, PAKISTAN

**Received:** June 27, 2025

**Accepted:** November 9, 2025

**Published:** December 2, 2025

**Copyright:** © 2025 Rahimipour Anaraki et al. This is an open access article distributed under the terms of the Creative Commons Attribution License, which permits unrestricted use, distribution, and reproduction in any medium, provided the original author and source are credited.

**Data availability statement:** There are ethical restrictions on sharing the dataset, as it contains potentially identifying information. These restrictions were imposed by

## Abstract

### Background

Emergency department (ED) overcrowding remains a significant national issue in Canada. To address this issue, SurgeCon, a quality improvement program, was implemented to enhance patient flow, improve communication, and reduce wait times. Despite their potential, interventions like SurgeCon lack evidence on real-world implementation and sustainability in high-pressure, resource-limited ED settings.

### Objective

This study explores the factors influencing the implementation of SurgeCon in four Canadian EDs using the Consolidated Framework for Implementation Research (CFIR) to identify facilitators and barriers.

### Methods

Data were collected over 2.5 years–before, during, and after SurgeCon implementation—in two rural and two urban EDs in Canada using a longitudinal qualitative research (LQR) design. Forty-two semi-structured interviews with physicians, nurses, and hospital managers were analyzed through inductive and deductive thematic analysis, guided by the CFIR framework.

### Results

Facilitators were predominantly associated with CFIR's Innovation Characteristics, particularly the perceived benefits of real-time data collection, workflow optimization,

the Health Research Ethics Board (HREB) of Newfoundland and Labrador, which approved this study. For data access requests, please contact the SurgeCon Team (SurgeCon@mun.ca).

**Funding:** Canadian Institutes of Health Research, Newfoundland and Labrador Provincial Government, Newfoundland and Labrador Health Services, and the Trinity Conception Placentia Health Foundation. The funders had no role in study design, data collection and analysis, decision to publish, or preparation of the manuscript.

**Competing interests:** I have read the journal's policy and the authors of this manuscript have the following competing interests: CP and PN are the co-founders of SurgeCon Innovations. Our team received funding for a project entitled "SurgeCon: An Emergency Department Surge Management Platform" from the Canadian Institutes of Health Research, Newfoundland and Labrador Provincial Government, Newfoundland and Labrador Health Services, and the Trinity Conception Placentia Health Foundation. PN has a royalty-sharing agreement with MOBIA Technology Innovations and the Newfoundland and Labrador Health Services. This does not alter our adherence to PLOS ONE policies on sharing data and materials.

and enhanced communication. However, barriers—mainly linked to outer setting (COVID-19 disruptions), inner setting (resource constraints and fragmented communication), and individual characteristics (leadership engagement and motivation)—outweighed these advantages.

## Conclusion

To strengthen adoption, this study proposes eight strategic action plans focusing on leadership commitment, automation, cross-departmental collaboration, feedback loops and change management strategies to maximize facilitators and address implementation barriers.

## Trial registration

ClinicalTrials.gov. NCT04789902. 10/03/2021.

## Introduction

Prolonged emergency department (ED) wait times are a global issue contributing to overcrowding. They frustrate patients, impact care perception, increase morbidity and mortality, heighten staff aggression, and lower retention and satisfaction [1–3]. Canadian ED wait time ranks among the highest in industrialized nations, posing a significant challenge [4].

To address these challenges, SurgeCon was developed in Newfoundland and Labrador, Canada, as a quality improvement program comprising three key interventions: a surge management platform, organizational restructuring, and a patient-centered approach to address logistical barriers to reduce wait times. Beyond reducing wait times, SurgeCon aims to enhance ED efficiency, improve patient care (e.g., enhancing comfort, navigation, and ED cleanliness), and strengthen communication amongst all clinicians and staff. [5–7].

However, implementing such a complex, non-drug, non-surgical intervention in dynamic environments like EDs requires careful planning. An implementation science framework is essential for guiding this process [8], ensuring systematic data collection, analysis, and interpretation [9]. The Consolidated Framework for Implementation Research (CFIR) is widely used to assess and guide the adoption of complex healthcare interventions. It consists of 38 constructs across five domains—intervention characteristics, outer setting, inner setting, individual characteristics, and process—helping identify facilitators and barriers [10,11].

Despite the promise of programs like SurgeCon, there is limited evidence on how such complex, multi-component interventions are implemented and sustained in real-world ED settings—particularly those facing high patient volumes, limited resources, and workforce constraints.

This study explores SurgeCon's implementation in four Canadian EDs using CFIR to assess facilitators and barriers throughout the process. To enhance the

effectiveness and sustainability of similar future interventions in dynamic healthcare settings, eight action plans were developed based on insights from healthcare providers and managers.

### SurgeCon: A quality improvement program

SurgeCon is a complex intervention with three key components: 1) SurgeCon– Software, a surge management platform automating surge capacity planning; 2) SurgeCon – Flow, a restructuring approach optimizing ED workflow; and 3) SurgeCon – Patient Centered Care, aimed at improving the patient experience.

The SurgeCon– Software predicts surge levels, automating workflows to assess and predict patient volumes, improves communication among staff, supports efficient patient flow through the ED, and helps alleviate overcrowding. Embedded within the ED nursing station, it provides real-time surge notifications via a smartphone app, email, and text. Algorithms calculate surge scores, triggering predefined responses. For instance, a score of 40 triggers Surge Level 5, prompting staff alerts, directing low-acuity patients to the waiting room, and notifying physicians about potential discharges [12].

SurgeCon–Flow improves ED efficiency through a staff flow course and management strategies for nurses and physicians. It emphasizes patient-centered care, collaboration between providers, and workflow optimization—prioritizing stable noncritical patients based on criteria beyond acuity, preserving nurse-monitored bedspaces, minimizing time to initial physician assessment, and streamlining patient care processes [6].

SurgeCon – Patient Centered Care enhances the patient experience by addressing factors affecting comfort, ease of navigation, and ED cleanliness, ensuring a more supportive environment.

## Methods

### Study design

This study employed a longitudinal qualitative research (LQR) design to assess the implementation of SurgeCon in four Canadian EDs over 2.5 years (pre-, during-, and post-implementation) [13]. LQR's unique focus is on understanding an experience or behavior over time, specifically addressing questions such as, "How did this change?", "What has stayed the same?", and "In what ways is this different?" [14].

Guided by CFIR, this study systematically examined: implementation of the intervention; mechanisms of impact—how participants engaged with and responded to the intervention; and context—organizational, political, and social influences affecting implementation. CFIR's five domains (Intervention Characteristics, Outer Setting, Inner Setting, Individual Characteristics, and Implementation Process) provided a structured lens to identify barriers and facilitators at different stages of implementation. Ethics approval for this study was obtained from the Health Research Ethics Authority (HREA) of Newfoundland & Labrador (HREB #2019.264). Participants provided informed consent prior to participation, with written consent obtained through signed consent forms.

### Study context

This study used a stepped-wedge cluster trial design to implement SurgeCon in Category A hospitals [6], all offering 24/7 physician coverage in their EDs. The rural intervention sites, each with 8 ED beds, had staffing of 6–10 physicians and 12–15 nurses. The urban sites included a large acute care facility (40 + beds) and a smaller one (15 beds) sharing a pool of 40 physicians. Staffing at urban EDs ranged from 55 to 70 nurses. The rural sites had specialist consultants in surgical, obstetrics, pediatrics, and internal medicine, while the urban sites offered comprehensive inpatient and outpatient services, including tertiary care [15].

### Data collection

From March 2021 to October 2024, we conducted semi-structured, in-depth interviews with 42 clinicians, including 29 from rural EDs and 13 from urban EDs, before, during, and after the implementation of SurgeCon. The group comprised

19 nurses, 12 physicians, 8 managers, 2 patient care facilitators, and 1 program coordinator, with their ED experience ranging from 1 to 32 years.

The interview questions were guided by CFIR [16] and incorporated insights from our team's clinical expertise. Eligible participants included healthcare providers and managers working at the four EDs. A purposive and snowball sampling strategy was employed to ensure diverse representation of key stakeholders, particularly the recruitment challenges posed by COVID-19, which limited stakeholder diversity. Participants were recruited through internal facilitators, such as nurse practitioners, who assisted with scheduling interviews, as well as by the research team during SurgeCon's training sessions. Additionally, team members personally reached out via email to encourage participation. Recruitment continued until data saturation was reached—defined as the point when no new themes or insights emerged from additional interviews—indicating a comprehensive understanding of the phenomenon and ensuring the depth and completeness of the findings. Throughout the data collection process, we continuously monitored saturation by comparing new data against existing codes and themes. Interviews with participants were conducted by qualitative researchers via phone, Zoom, and in person.

### Data analysis

A thematic analysis was conducted using an inductive-deductive hybrid approach, capturing both emerging themes and predefined CFIR constructs. Inductive coding revealed eight action plans to address the challenges and barriers identified by healthcare providers. Deductive coding systematically mapped codes onto CFIR's five domains to structure the analysis. For instance, codes like "love to have some feedback," and "what benefit SurgeCon is for our department" were mapped to CFIR's Inner Setting: Access to Knowledge and Information, while "biggest takeaway is flow center," "SurgeCon helps us organize ourselves," and "positive aspects around flow center" were mapped to CFIR's Intervention Characteristics: Relative Advantage, demonstrating perceived benefits of SurgeCon. Fig 1 presents the 17 CFIR constructs that emerged most prominently from the interview data. Two researchers independently coded transcripts, resolving discrepancies through consensus discussions to enhance reliability. Peer debriefing, investigator triangulation, and validation across professional roles (physicians, nurses, managers) ensured credibility and trustworthiness [17,18].

## Findings

In our analysis, 17 out of 38 CFIR constructs were examined (Fig 1). Notably, there were no differences in identified barriers and facilitators between rural and urban EDs. The following sections delve into these factors, providing illustrative quotes to enrich the discussion (Table 1).

### 1. Innovation domain

**Innovation source.** There was skepticism about the effectiveness of bringing in an external company to address departmental issues. This skepticism was rooted in a deeper cultural attitude where the ED staff felt that their intimate knowledge of daily operations and challenges was overlooked.

*"lot of people felt like, 'Well, why do we need an outside company? Why don't they just speak to the staff that actually works there to see how they could fix it?' We knew what needed to be fixed but I kind of felt amused as to why did an external entity do it when they didn't ask the people that worked in a department first."*

**Innovation design.** SurgeCon-Software's lack of automation increased the burden on staff, who were already overwhelmed by high patient volumes and static staffing levels. While data entry only took 1–3 minutes every 4 hours, the challenge was maintaining consistency due to the added workload.

*"Our staff are pretty good, but again when you need to put in the SurgeCon or you know we try to do it every so often, but those times when it's at peak business, it's often difficult to put it in."*

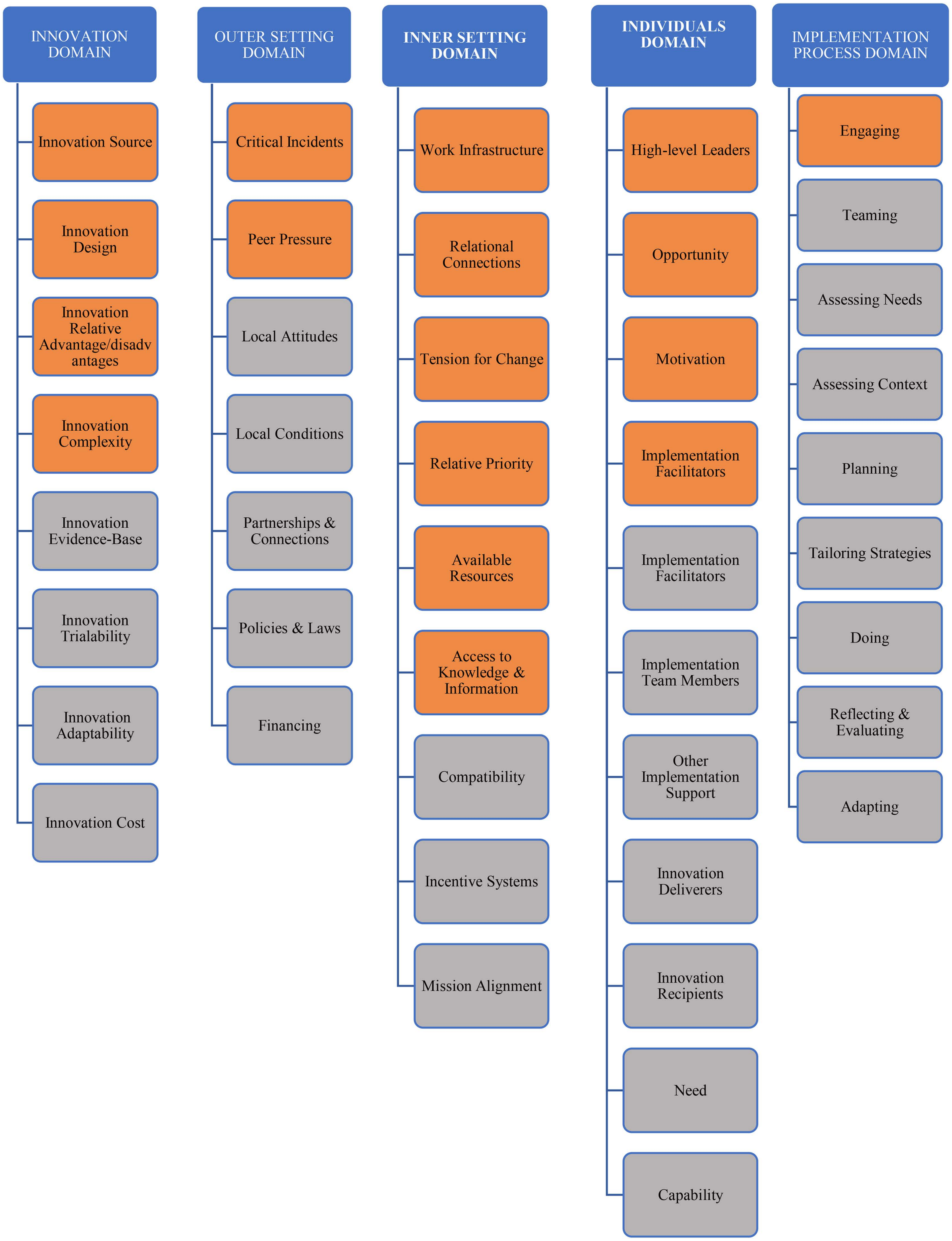


**Fig 1. Overview of the Consolidated Framework for Implementation Research: The analyzed data were categorized into 17 constructs (represented by the orange boxes).** Data for the remaining constructs (represented by the gray boxes) were not available.

https://doi.org/10.1371/journal.pone.0337389.g001

Table 1. Facilitators and Barriers of SurgeCon Intervention using the Consolidated Framework for Implementation Research.

| I. INNOVATION DOMAIN | Barriers | Facilitators |
|---|---|---|
| **Innovation Source** | - Resistance to external entities and outsiders' plans for change | |
| **Innovation Design** | - Lack of automation | |
| **Innovation Relative Advantage/ disadvantages** | | - Real-time data for managing patient care<br>- Streamlines communication between front-line staff and managers<br>- Successful implementation of patient flow |
| **Innovation Complexity** | | - Simplicity and user comfort of the tool |
| **II. OUTER SETTING DOMAIN** | | |
| **Critical Incidents** | - Challenges due to COVID-19 impact<br>- Frequent policy changes during COVID-19 | |
| **Peer Pressure** | | - Direct Observation of Successful Practices in Other Hospitals Fosters Healthy Competition |
| **III. INNER SETTING DOMAIN** | | |
| **Work Infrastructure** | - Shortage of human resources | |
| **Relational Connections** | - Poor communication and fragmented relationships between nurses and physicians | |
| **Tension for Change** | - Gap between identifying problems and implementing effective solutions.<br>- Low morale and burnout due to the pandemic | - Tension to improve wait time and workflow |
| **Relative Priority** | - Competing priorities such as patient care | |
| **Available Resources** | - Lack of physical space and rooms | |
| **Access to Knowledge & Information** | - lack of clear communication regarding the impact of SurgeCon on their department | - Biggest takeaway from the SurgeCon is training |
| **IV. INDIVIDUALS DOMAIN** | | |
| **High-level Leaders** | - An incomplete comprehension of project's full value<br>- Minimal managerial commitment | - Managers and leaders generally expressed interest in the project |
| **Implementation Facilitators (Champions)** | - Not enough champions | - Champions played a pivotal role in the successful implementation |
| **Opportunity** | - Excessive workload and lack of time | |
| **Motivation** | - Lack of care and responsibility | |
| **V. IMPLEMENTATION PROCESS DOMAIN** | | |
| **Engaging** | - Lack of engagement from other hospital departments | |

https://doi.org/10.1371/journal.pone.0337389.t001

**Relative advantage.** Participants identified both advantages and disadvantages of SurgeCon-Software. Participants emphasized the importance of real-time data for managing patient care, and noted that SurgeCon-Software captured an accurate picture of the department's status.

*"I like that it like takes all the data that we're putting in and then like presents like a level of, I guess, acuity in the department. It's like an accurate, I find it's an accurate representation of what's actually going on."*

The automated email notifications sent to managers when the SurgeCon-Software level exceeds three. This feature was considered a valuable advantage of SurgeCon-Software. Participants appreciated how this feature streamlines communication between frontline staff and managers, communicating the workload and operational challenges faced by frontline staff.

*"I liked the part where we could update the board and that it was a direct link, especially once you got above level three, that my manager would get an email. It would save me having to phone him, text him, call him, try to track him*

*down, and he would check in and say, okay, I see you're in SurgeCon level, whatever, and then he would make the necessary moves or try to with the other units to get our patients out. So I felt like it was a good communication tool"*

The successful implementation of SurgeCon-Flow attributed to the strong "buy-in" from triage nurses, who were the primary beneficiaries of the new system. By reducing patient wait times and frustration, it improved their work environment, allowing them to focus more effectively on patient triage and ensuring smoother patient flow.

*"One of the big buy-ins for us around SurgeCon was when we started doing the flow center and started using some of the techniques. The triage nurses were the people who really bought into it the most because it impacted them the most, largely due to the fact they didn't have as many patients waiting in the waiting room, they didn't have many upset patients"*

**Innovation complexity.** Participants noted that the basic tasks involved in using SurgeCon-Software, such as entering data about bed occupancy and patient wait times, were relatively simple and did not pose significant challenges for most users. The process of updating these metrics was quick and straightforward, taking only a few minutes to complete.

*"But the rest is pretty simple because it's only like how many beds are occupied in the unit. How many people are waiting to be seen and their triage level, so that's pretty easy. It takes about like 5 or 6 minutes to do the whole thing. So, it's not hard; no."*

## 2. Outer setting

**Critical incidents.** COVID-19 pandemic not only created immediate operational challenges but also exacerbated issues related to health human resources, which continued to affect the project's success. Frontline staff faced a confluence of challenges, including extreme exhaustion from high workloads, frustration from dealing with constant crises, and burnout from the prolonged stress of the pandemic.

*"With this pandemic, there's constant policy changes, procedure changes, and they're frustrated with it. So, if you want to bring in something else, even though it's going to help them a lot of times- they're resistant because it's just something else on their 'To Do List' and they don't want to be bothered with having to learn something else."*

**Peer pressure.** Participants noted that direct observation of successful practices drove change. By inviting staff from site A to observe site B's success, the team aimed to inspire similar initiatives, using social dynamics to motivate adoption.

*"Seeing is believing. So I think what we're trying to do is trying to get some staff from [site B] to come to the [site A] to see how well it works and maybe what that might look like at [site B]. So I think, you know, that might be kind of a bit of peer pressure, I guess, to some extent, or maybe some healthy competition or whatever you might want to call it"*

## 3. Inner setting

**Work infrastructure.** The absence of essential resources, such as human resources, significantly hindered the implementation and maintenance of SurgeCon Flow. At one site, the absence of a manager significantly disrupted efficiency and created challenges for the staff. Staff struggled due to the lack of leadership and guidance, and substitute managers, who were already stretched thin with their own departmental duties, were unable to provide the necessary support.

*"I think that's a big issue as our manager has been sick for a while and so the other managers in the hospital are covering for him and they're busy with their So, they're busy with their own department. So then. It's very hard for them to come here and help us sort out what's wrong here."*

One of the participants emphasized a critical shortage of staff for data entering, relying heavily on a single individual tasked with inputting information while also managing patient transport throughout the hospital. This individual had limited availability, particularly during peak ED hours when accurate data input was most crucial.

*"Big barrier for us was a lot of days that the data wasn't tracked. So, we had our [staff] actually as the main person to implement the input information and [they are] all over the hospital; So [they] not always in the department. I don't think we fully captured those numbers because at those times we didn't have someone to implement all the data to input it in the system"*

**Relational connections.** Effective and frequent communication between clinicians, especially between physicians and nurses, was necessary for the successful execution of implementation activities for all three components of SurgeCon. Challenges such as poor communication and fragmented relationships between nurses and physicians, as well as a lack of teamwork, emerged as significant barriers to implementing and maintaining SurgeCon.

*"We do not sit down at the same table. There are family practice meetings, there are student emergency doc meetings and then, there are nursing meetings; you are not sat at the same table. So, I cannot realistically know, feel nor empathize with anybody else's needs if I am not even aware of them. We are never really made aware of that stuff."*

**Tension for change.** A critical challenge in the EDs was the gap between identifying problems and implementing effective solutions. This disconnect points to a broader cultural issue within the department: a lack of tension for change. In EDs, alert levels were crucial for signaling the severity of the situation and mobilizing appropriate responses. At alert level 5, management should take urgent actions to resolve overcrowding by finding additional beds, involving more doctors, and facilitating patient discharges. However, the problem went beyond SurgeCon. Although SurgeCon-Software provided clear indicators of the ED's status and sent out notifications about the crisis, there appeared to be a gap between recognizing the problem and implementing solutions.

*"Surge Level 5, trying to get that pushed or escalated upward and into our conversations of flow throughout the hospital and creating flow. So, you know, if we're, you know, we're working at something or inputting data in things like people want to see an action or a result. So, without action or results, it becomes a little, people feel that it may be a little, that the work they did or the data they entered might be, is a little bit of a futile"*

Low morale and burnout due to the pandemic among staff were significant obstacles to motivation. High business demands and insufficient resources were causing exhaustion and dissatisfaction, which in turn hindered staff's willingness to support or engage in new initiatives.

*"I mean morale in the past few years… it's not in a good place and I think it's because of the increased business, and staff feel like they're burning out, so it's not that they don't do a good job. We need more resources."*

**Relative priority.** Participants noted that while entering data into SurgeCon Software was not time-consuming, it competed with the urgent demands of patient care in the ED. SurgeCon-Software struggled to gain traction due to competing priorities across departments and the overwhelming workload and staffing shortages, which led to its deprioritization.

*"I mean, it doesn't take long to fill it out- it's just actually like… that's not a priority when there's patients who need immediate care, right? So I don't know if, like, if a ward clerk could do it or like someone in reception, had a minute to come back and fill it out. Doesn't necessarily have to be a nurse, right"*

**Available resources.** Participants frequently identified insufficient admission space (e.g., not enough beds), a lack of physical space, and rooms in EDs as major contributors to backlogs and overcrowding. These issues significantly impacted implementation of SurgeCon-Flow as they impact patient admissions, transfers, discharges, and the restructuring of ED workflows. Despite being aware of the need to move patients and make coordinated efforts with other units, the lack of available beds often impeded effective patient flow management.

*"I will get an alert on my phone that we are at SurgeCon 5. So, then I'm aware that it's very busy and that we need to move patient's…but it is the bed availability."*

**Access to knowledge and information.** One of the key strategies used to enhance engagement and involvement in the SurgeCon-Flow implementation process was ED staff training, generally highly regarded by participants.

*"I think the biggest takeaway from the SurgeCon work for me is the flow center and a lot of the training that we did with the nurses and the staff to create like a flow center or a surge center was quite good"*

The opportunity to evaluate EDs' flow was a significant advantage, fostering a mindset geared toward enhancement and efficiency.

*"I think the general concepts that SurgeCon aims to address flow, thinking of different ways to see patients when you have a big block department, I think those lessons still hold true and still. Our physicians did take some info from the*

*[training] sessions in that regard, and they've used that this like, not necessarily have the data input it to support the actual SurgeCon app or application- whatever, but I do think that was an advantage and that, you know it was good to kind of analyze our department take a look at our flow and look at ways that we could improve it."*

However, one of the champions mentioned a lack of clear communication regarding the impact of SurgeCon on their department and emphasized the need for data-driven feedback to validate the program's success. They stressed that effective communication of data and regular updates were crucial for demonstrating the program's benefits and justifying further support.

*"I would like to know personally what SurgeCon has done for us in [place]. Like, are our numbers showing that we need more staff? Do we have enough people? Like, what is it exactly that we're extracting from this data, and how is it helping us? I would love to know that, some feedback"*

## 4. Individuals domains

**High-level leaders.** Participants emphasized the importance of managers being actively involved and viewing themselves as key contributors to the change initiative.

*"they [managers] impact greatly, but again, no different. They have to be a part of the change management process, right, so they have to be bought on board and see themselves in the change, and that part, I think, needs more work"*

While managers generally expressed interest in the project and demonstrated a degree of engagement, there was a notable difference in site readiness and uncertainty regarding the extent to which EDs would embrace the project.

*"I think leadership, depending on the site, was acceptable. We have different levels, different sites where leaderships are more accepting. But overall, I think we were accepting, but at different sites, there was more levels of readiness"*

Consistent managerial commitment in the EDs is essential for SurgeCon's effectiveness. When its action plans and notifications are ignored, no improvements occur. Although SurgeCon provides strategies to manage patient flow—such as reallocating staff or transferring patients—its success relies on managers executing key measures like increasing staffing during peak times, adding physician coverage, or creating more admission space.

*"The only thing is, it doesn't change the process like I find very rarely. So, I'll say- this morning, for example. I entered in all the information [and] updated the SurgeCon when we came on shift. So, we're now at level 5, we have an ICU admission. We have admissions waiting for medicine. We had two people on isolation. OK, so that's fine… But nothing changes. You don't get like- when my manager is not calling to see how we can move people"*

One of the participants highlighted the active involvement of their nursing manager in ensuring that not only nurses but also physicians received the SurgeCon Flow and SurgeCon PCC training. To facilitate this training, the team adjusted their schedules, demonstrating a commitment to staff development and collaboration.

*"Our nursing manager was fairly involved in making sure that all of our nurses had training and on the chief for site and you know, I think we managed to get almost all of our physician body there, not quite everybody, but most people over the course of the day and we made changes to our schedule to allow that to happen."*

**Implementation facilitator.** Site champions played a pivotal role in the successful implementation of SurgeCon-Flow. However, one person as a champion was not effective; the more champions involved in the solution, the higher the likelihood of its success.

*"So, you know, it's like everything a solution. If one person knows the solution to idea, but a group doesn't work on to it, you know, that solution might not work out, but not necessarily say it's a bad solution. It's just that there's just not enough people championing the solution. So, you know, the more champions, the better"*

**Opportunity.** To implement the new system, it was crucial that healthcare providers not only be available and had enough time to participate in staff flow training, but also consistently enter and update data in SurgeCon Software. However, most participants noted a shortage of medical staff. As a result, core nurses had to manage both patient care and data entry, while travel nurses, lacking knowledge of the SurgeCon Software, could not assist with these critical tasks.

*"like they know that it exists but usually whoever's in charge is responsible for it. And always like, if you're- there's one core nurse… So, if I'm working a shift with two travel nurses, I'm the one who's in charge because I'm responsible for that. We haven't been putting travel nurses in-charge, because it just tends to be. they don't know the system, I guess as well as the core staff do. So, having a shortage of staff- and then on a really busy day, if you're short staffed, you don't you just don't have time to do SurgeCon,"*

**Motivation.** Lack of motivation and a desire for change were identified as crucial barriers to maintaining all aspects of SurgeCon (Software, Flow, PCC), as participants expressed concerns such as "nobody seems to care about it" and "I feel like nobody is policing it." This lack of motivation led to a deficiency in long-term planning and collaboration, which were essential for leveraging data to support meaningful changes. Despite having the necessary data to drive improvements, there was a perceived lack of care and responsibility, which undermined the effectiveness of SurgeCon and hindered progress.

*"I don't think they could care less, and everybody is crying that they have no staff, so they don't care that emerge is overwhelmed. They- I mean we need a collaborative effort to open those beds, and it's because it's bottlenecking the emergency department, and it's like every day I just can't be in anybody's hands. So they don't really care about SurgeCon numbers because I brought it up, you know, we're SurgeCon level 5. Nobody cares"*

### 5. Process domains

**Engaging.** Participants expressed concerns about the limited engagement of other hospital departments in the SurgeCon Software and SurgeCon Flow. They highlighted the need for active involvement across all departments for the intervention to succeed, emphasizing that improving ED patient flow should be a hospital-wide priority. A key challenge was the software's lack of integration into daily operations beyond the ED, with insufficient follow-up actions from other units, such as patient transfers and doctor engagement.

*"Had a lot of difficulties with implementation of SurgeCon and not so much from the front line, but more, I guess, overall adaptation of, making SurgeCon work beyond the emergency department. So, you know, if we do have, we're in a surge level, making sure that, you know, we can create actions that will help flow and whatnot."*

**Actionable Plans for Enhancing SurgeCon Implementation.** While theoretical frameworks like CFIR offer a structured approach to analyzing implementation challenges, real-world success relies on practical, provider-driven solutions. This study identifies eight key action plans, informed by participants' feedback, to address barriers and enhance the process and sustainability of the intervention (Table 2).

## Discussion

Implementing a complex intervention like SurgeCon in EDs, especially in uncertain times, proved to be highly challenging. A key insight from this study is that the success of SurgeCon was not determined by the complexity or usability of the software itself, but by the organizational and contextual factors. While the innovation posed minimal technical challenges, contextual and organizational barriers—such as work infrastructure, relational dynamics, and insufficient involvement from high-level leaders —played a much more significant role in shaping the outcomes. This underscores that in dynamic settings like emergency departments, the readiness of the organization and the alignment of leadership at multiple levels are often more critical to implementation success and sustainment than the features of the innovation itself.

The innovation domain emerged as the most distinguishing construct. According to Abbott et al. [19], the implementation of technological innovations in complex healthcare environments poses significant challenges, especially when such interventions disrupt existing organizational structures and clinical workflows. However, SurgeCon Software posed minimal challenges, with quick and straightforward metric updates. Participants recognized SurgeCon's real-time data and improved communication between staff and management as a clear advantage. This aligns with Weir et al. [20], who emphasized that demonstrating the relative advantage of innovations is crucial for adoption, especially in improving

**Table 2. Data Driven Action Plans to Improve the Effectiveness and Sustainability of SurgeCon in EDs.**

| Actions | Explanation | Exemplar Quotes | Barriers to Improvement through this Action Plan | CFIR Domains |
|---|---|---|---|---|
| **1-Expand Champion Involvement** | SurgeCon would benefit from having champions across various hospital departments to bridge gaps and better address challenges | *"Maybe some champions outside of the emergency department. So, somewhere else where somebody, somebody sees value in it and understands it outside of the program. So we have, when we did the search on say training and those types of things, everybody in that room understands the language, understands the problem."* | - Lack of clear communication regarding the impact of SurgeCon on their department<br>- Not enough champions<br>- Lack of care and responsibility | - **Inner Setting Domain**<br>- **Individuals Domain** |
| **2-Foster Collaboration Across Departments** | Effective patient flow and resource management require cross-departmental collaboration, not just reliance on the ED | "*What we need to do is a collaborative effort issue made between the emergency department and the other unit, so that we can get our beds open and get some staff on it collaboratively instead of piling everything on one unit"* | - Limited engagement and collaboration of departments outside the ED | - **Implementation Process Domain** |
| **3-Enhance Training for Team Leads** | Training team leads in all hospital units on SurgeCon's functions is essential for improving the patient transfer process | *"I feel that we need more training again for the team lead on the other units because they do get the emails and I feel that SurgeCon should help us facilitate some of these transfers without making 7,000 phone calls"* | - Resistance to external entities and outsiders' plans for change<br>- Limited engagement and collaboration of departments outside the ED<br>- An incomplete comprehension of project's full value | - **Innovation Domain**<br>- **Implementation Process Domain**<br>- **Individuals Domain** |
| **4-Automate Key Processes** | Automating SurgeCon would improve efficiency by focusing on decision-making instead of manual data entry | *"I think if we had some more ease of, I think if it wasn't a manual process, maybe something that could be a bit more automated, that might help as well"* | - Lack of automation<br>- Excessive workload and lack of time | - **Innovation Domain**<br>- **Inner Setting Domain** |
| **5-Use Peer Observation to Drive Adoption** | Using peer observation can help demonstrate SurgeCon's effectiveness | *"Seeing is believing. So I think what we're trying to do is trying to get some staff from [site B] to come to the [site A] to see how well it works and maybe what that might look like at [site B]. So I think, you know, that might be kind of a bit of peer pressure, I guess, to some extent, or maybe some healthy competition or whatever you might want to call it"* | - Poor communication and fragmented relationships between nurses and physicians<br>- Low morale and burnout due to the pandemic<br>- An incomplete comprehension of project's full value | - **Inner Setting Domain**<br>- **Individuals Domain** |
| **6-Ensure Decisive Collaborative Leadership** | Strong management is necessary for making quick, collaborative decisions in high-alert situations | *"If your emergency room is in alert level 5, then management should figure out a way to resolve that as best as possible. They should meet together, see where we can get beds, see what doctors should get involved, see who they can discharge"* | - An incomplete comprehension of project's full value<br>- Gap between identifying problems and implementing effective solutions<br>- Lack of physical space and rooms | - **Individuals Domain**<br>- **Inner Setting Domain** |
| **7-Develop a Comprehensive Change Management Plan** | A solid change management plan is crucial for preparing departments for SurgeCon's implementation and ensuring smooth adoption | *"The tool may be fine, but there has to be more done from a change perspective to introduce these new concepts… I suggest that, obviously, before these tools are implemented, there has to be a robust change management work done, right? That probably would be the way to get around, get over some of the barriers"* | - Shortage of Human Resources<br>- Poor communication and fragmented relationships between nurses and physicians<br>- Gap between identifying problems and implementing effective solutions<br>- Lack of physical space and rooms<br>- Excessive workload and lack of time | - **Inner Setting Domain** |
| **8-Establish Feedback Loops** | Real-time data feedback about performance can significantly boost motivation and participation | *"Because our numbers, I feel, have been extremely high for a long time, but I haven't gotten any data or any feedback, or, like, I don't really know how we're doing compared to other areas. We might be doing better than I think, and we could be doing worse. Like, I don't know. I have no way of knowing"* | - Lack of clear communication regarding the impact of SurgeCon on their department<br>- An incomplete comprehension of project's full value<br>- Lack of care and responsibility | - **Inner Setting Domain**<br>- **Individuals Domain** |

https://doi.org/10.1371/journal.pone.0337389.t002

communication. Despite these advantages, SurgeCon's manual data entry design was a barrier, supporting Lam et al. [21], who noted that manual input increases cognitive load and leads to user fatigue.

Within the outer setting domain, external factors, especially the COVID-19 pandemic, played a critical role in influencing SurgeCon's implementation (Software, Flow, PCC). The pandemic diverted resources and attention from the project, limiting its impact and causing delays, a barrier also reported by Hennein et al. [22], who documented how external crises disrupted healthcare initiatives. Conversely, peer dynamics and competition among EDs fostered positive engagement. Seeing SurgeCon's success in other EDs motivated staff and drove adoption, highlighting the power of social influence. This contrasts with Warner et al. [23], who found peer pressure less effective in decentralized settings, suggesting stronger influence in closely networked EDs.

Within the inner setting domain, staff shortages were a recurring theme, significantly hindering the implementation of SurgeCon. This aligns with King et al. [24] and McGuier et al. [25], who emphasize that robust infrastructure, including adequate staffing and communication systems, is vital for sustaining new interventions. McGuier et al. [25] emphasize that strong relational connections and interdisciplinary teamwork are critical for successful implementation. This aligns with our findings, where weak interdepartmental relationships—particularly between physicians and nursing staff—were identified as a barrier. Ahumada-Canale et al. [26] and Shin et al. [27] observed that competing priorities often shift focus from new initiatives, with ED staff prioritizing patient care. Finally, limited admission space exacerbated SurgeCon's implementation challenges, as highlighted by Lan et al. (2019) and Hennein et al. [22].

Within the individuals domain, frontline staff champions were key to gaining buy-in and overcoming resistance, emphasizing the need for strong advocates. This aligns with Bonawitz et al. [28], who highlight champions as crucial for successful healthcare implementation. However, the findings show that SurgeCon lacked champions beyond the ED, limiting its perceived importance. Bonawitz et al. [28] and Miech et al. [29] stress that champions with broad influence and leadership are key to promoting innovation. The absence of champions from other departments, like administration or IT, hindered SurgeCon's broader integration. Additionally, in EDs where SurgeCon's insights were not acted upon by the managers, staff became disillusioned, feeling that their efforts in data entry did not lead to meaningful change.

The process domain revealed that effective communication and the active engagement of all relevant stakeholders across all hospital departments were crucial for maximizing SurgeCon's impact. This aligns with Breimaier et al. [10], who emphasized that the engagement of all stakeholders is essential to ensure alignment with an intervention's objectives and to maintain staff motivation.

Eight actionable plans were extracted based on healthcare providers' suggestions. Engaging providers ensures the development of practical, context-aligned action plans, thereby improving intervention success [30,31]. Based on the findings of this study, future interventions would benefit from expanding champion involvement, fostering cross-departmental collaboration, enhancing training, leveraging peer influence, ensuring decisive leadership, developing a comprehensive change management plan, and establishing feedback loops.

## Conclusion

This study offers a comprehensive study of SurgeCon's implementation in Canadian EDs guided by the CFIR. The findings highlight SurgeCon's potential to enhance patient flow and management through monitoring wait times, improving data collection, and establishing a flow center.

This study highlights that while SurgeCon holds promise in improving ED operations, its success is contingent on overcoming contextual barriers, particularly leadership engagement, staff capacity, and cross-departmental integration. The findings emphasize that automation, clear leadership structures, and sustained interdepartmental collaboration are critical for ensuring the long-term sustainability of ED innovations. Future research should explore automated data collection solutions and examine the action plans to optimize implementation outcomes.

This study has limitations. This study focused on SurgeCon implementation in four Canadian EDs, which may not fully apply to other healthcare settings. However, we emphasize the importance of transferability and encourage future research to explore how these insights might apply in similar contexts. Additionally, the study was conducted during a dynamic period, with the COVID-19 pandemic causing rapid changes to healthcare policies, which may have influenced the barriers and facilitators identified. Furthermore, only 17 CFIR constructs emerged from the data. The remaining constructs were not grounded in the data due to their limited relevance to the participant group. Most participants were healthcare providers which restricted insight into certain CFIR constructs—particularly those related to innovation cost and outer setting factors (e.g., financing, policies, and regulations). In other words, while our study provides in-depth perspectives from emergency department staff and managers, it does not reflect perspectives from other key hospital stakeholders, such as IT and administrative personnel.

By applying the CFIR framework, this study provides a deeper understanding of how specific constructs interact to shape the implementation of complex interventions in emergency care. In doing so, it extends CFIR's applicability to dynamic, resource-constrained settings and offers valuable theoretical insights for future implementation science research in acute care contexts.

Taken together with the practical findings, this study contributes valuable insights into the complex realities of implementing innovation in emergency care. Addressing contextual barriers and embedding implementation strategies into the organizational fabric will be essential for future ED transformation efforts.

For hospital administrators and policymakers, this study offers several actionable recommendations, from appointing cross-functional champions to lead implementation efforts to investing in automation to reduce manual workload. Implementing such strategies can significantly enhance the adoption and sustainability of complex interventions like SurgeCon.

Looking ahead, as EDs continue to face pressures from overcrowding and staffing shortages, innovations grounded in implementation science will be essential. This study adds to the growing body of work aimed at bridging the gap between innovation design and real-world application, offering a roadmap for more effective and sustainable emergency care.


## Acknowledgments

We sincerely thank our patient partners for their invaluable contributions to the development and successful completion of this manuscript. We also appreciate all team members' advice and support.


## Author contributions

**Conceptualization:** Nahid Rahimipour Anaraki, Meghraj Mukhopadhyay, Christopher Patey, Paul Norman, Jennifer Jewer, Holly Etchegary, Oliver Hurley, Anna Walsh, Dorothy Mary Senior, Shabnam Asghari.

**Data curation:** Nahid Rahimipour Anaraki, Christopher Patey, Shabnam Asghari.

**Formal analysis:** Nahid Rahimipour Anaraki, Jennifer Jewer, Holly Etchegary, Shabnam Asghari.

**Funding acquisition:** Christopher Patey, Paul Norman, Shabnam Asghari.

**Investigation:** Shabnam Asghari.

**Methodology:** Nahid Rahimipour Anaraki, Holly Etchegary, Shabnam Asghari.

**Project administration:** Christopher Patey, Paul Norman, Oliver Hurley, Anna Walsh, Shabnam Asghari.

**Resources:** Christopher Patey, Paul Norman, Shabnam Asghari.

**Software:** Christopher Patey, Paul Norman, Shabnam Asghari.

**Supervision:** Christopher Patey, Shabnam Asghari.

**Validation:** Shabnam Asghari.

**Visualization:** Shabnam Asghari.

**Writing – original draft:** Nahid Rahimipour Anaraki, Meghraj Mukhopadhyay, Christopher Patey, Paul Norman, Shabnam Asghari.

**Writing – review & editing:** Nahid Rahimipour Anaraki, Meghraj Mukhopadhyay, Christopher Patey, Paul Norman, Jennifer Jewer, Holly Etchegary, Oliver Hurley, Anna Walsh, Dorothy Mary Senior, Peizhong Peter Wang, Shabnam Asghari.

## References


1. Kayuni J, Dordunoo D, Mallidou M, Mohan M, Marquard P. Exploring delay points at the emergency department. CJEN. 2024;47(1):14–9. https://doi.org/10.29173/cjen158
2. Ackroyd-Stolarz S, Read Guernsey J, Mackinnon NJ, Kovacs G. The association between a prolonged stay in the emergency department and adverse events in older patients admitted to hospital: a retrospective cohort study. BMJ Qual Saf. 2011;20(7):564–9. https://doi.org/10.1136/bmjqs.2009.034926 PMID: 21209130
3. Shah S, Patel A, Rumoro DP, Hohmann S, Fullam F. Managing patient expectations at emergency department triage. Patient Experience Journal. 2015;2(2):31–44. https://doi.org/10.35680/2372-0247.1090
4. Commonwealth Fund. Mirror, mirror 2021: Reflecting poorly: Health care in the US compared to other high-income countries. 2021.
5. Anaraki NR, Jewer J, Hurley O, Mariathas HH, Young C, Norman P, et al. Implementation of an ED surge management platform: a study protocol. Implement Sci Commun. 2022;3(1):21. https://doi.org/10.1186/s43058-021-00247-1 PMID: 35236510
6. Mariathas HH, Hurley O, Anaraki NR, Young C, Patey C, Norman P, et al. A Quality Improvement Emergency Department Surge Management Platform (SurgeCon): Protocol for a Stepped Wedge Cluster Randomized Trial. JMIR Res Protoc. 2022;11(3):e30454. https://doi.org/10.2196/30454 PMID: 35323121
7. Patey C, Norman P, Araee M, Asghari S, Heeley T, Boyd S, et al. SurgeCon: Priming a Community Emergency Department for Patient Flow Management. West J Emerg Med. 2019;20(4):654–65. https://doi.org/10.5811/westjem.2019.5.42027 PMID: 31316707
8. Campbell M, Fitzpatrick R, Haines A, Kinmonth AL, Sandercock P, Spiegelhalter D, et al. Framework for design and evaluation of complex interventions to improve health. BMJ. 2000;321(7262):694–6. https://doi.org/10.1136/bmj.321.7262.694 PMID: 10987780
9. Grootjans SJM, Stijnen MMN, Hesdahl-De Jong I, Kroese MEAL, Ruwaard D, Jansen MWJ. Implementation of an integrated community approach in deprived neighbourhoods: a theory-based process evaluation using the Consolidated Framework for Implementation Research (CFIR). Scand J Public Health. 2024;52(7):838–48. https://doi.org/10.1177/14034948231199804 PMID: 37726916
10. Breimaier HE, Heckemann B, Halfens RJG, Lohrmann C. The Consolidated Framework for Implementation Research (CFIR): a useful theoretical framework for guiding and evaluating a guideline implementation process in a hospital-based nursing practice. BMC Nurs. 2015;14:43. https://doi.org/10.1186/s12912-015-0088-4 PMID: 26269693
11. Damschroder LJ, Aron DC, Keith RE, Kirsh SR, Alexander JA, Lowery JC. Fostering implementation of health services research findings into practice: a consolidated framework for advancing implementation science. Implement Sci. 2009;4:50. https://doi.org/10.1186/1748-5908-4-50 PMID: 19664226
12. Jewer J. Investigating a Work System Approach to Implement an Emergency Department Surge Management System: Case Study. J Med Internet Res. 2022;24(8):e37472. https://doi.org/10.2196/37472 PMID: 36006684
13. Calman L, Brunton L, Molassiotis A. Developing longitudinal qualitative designs: lessons learned and recommendations for health services research. BMC Med Res Methodol. 2013;13:14. https://doi.org/10.1186/1471-2288-13-14 PMID: 23388075
14. Saldaña J. Longitudinal qualitative research. Rowman & Littlefield Publishers. 2003.
15. Anaraki NR, Mukhopadhyay M, Jewer J, Patey C, Norman P, Hurley O, et al. A qualitative study of the barriers and facilitators impacting the implementation of a quality improvement program for emergency departments: SurgeCon. BMC Health Serv Res. 2024;24(1):855. https://doi.org/10.1186/s12913-024-11345-w PMID: 39068432
16. Damschroder LJ, Reardon CM, Widerquist MAO, Lowery J. The updated Consolidated Framework for Implementation Research based on user feedback. Implement Sci. 2022;17(1):75. https://doi.org/10.1186/s13012-022-01245-0 PMID: 36309746
17. Lincoln YS, Guba EG. Naturalistic inquiry. Newberry Park, CA: Sage; 1985.
18. Thurmond VA. The point of triangulation. J Nurs Scholarsh. 2001;33(3):253–8. https://doi.org/10.1111/j.1547-5069.2001.00253.x PMID: 11552552
19. Abbott PA, Foster J, Marin H de F, Dykes PC. Complexity and the science of implementation in health IT--knowledge gaps and future visions. Int J Med Inform. 2014;83(7):e12-22. https://doi.org/10.1016/j.ijmedinf.2013.10.009 PMID: 24444700
20. Weir NM, Newham R, Dunlop E, Bennie M. Factors influencing national implementation of innovations within community pharmacy: a systematic review applying the Consolidated Framework for Implementation Research. Implement Sci. 2019;14(1):21. https://doi.org/10.1186/s13012-019-0867-5 PMID: 30832698

21. Lam H, Quinn M, Cipriano-Steffens T, Jayaprakash M, Koebnick E, Randal F, et al. Identifying actionable strategies: using Consolidated Framework for Implementation Research (CFIR)-informed interviews to evaluate the implementation of a multilevel intervention to improve colorectal cancer screening. Implement Sci Commun. 2021;2(1):57. https://doi.org/10.1186/s43058-021-00150-9 PMID: 34059156
22. Hennein R, Ggita J, Ssuna B, Shelley D, Akiteng AR, Davis JL, et al. Implementation, interrupted: Identifying and leveraging factors that sustain after a programme interruption. Glob Public Health. 2022;17(9):1868–82. https://doi.org/10.1080/17441692.2021.2003838 PMID: 34775913
23. Warner G, Lawson B, Sampalli T, Burge F, Gibson R, Wood S. Applying the consolidated framework for implementation research to identify barriers affecting implementation of an online frailty tool into primary health care: a qualitative study. BMC Health Serv Res. 2018;18(1):395. https://doi.org/10.1186/s12913-018-3163-1 PMID: 29855306
24. King DK, Shoup JA, Raebel MA, Anderson CB, Wagner NM, Ritzwoller DP, et al. Planning for Implementation Success Using RE-AIM and CFIR Frameworks: A Qualitative Study. Front Public Health. 2020;8:59. https://doi.org/10.3389/fpubh.2020.00059 PMID: 32195217
25. McGuier EA, Kolko DJ, Aarons GA, Schachter A, Klem ML, Diabes MA, et al. Teamwork and implementation of innovations in healthcare and human service settings: a systematic review. Implement Sci. 2024;19(1):49. https://doi.org/10.1186/s13012-024-01381-9 PMID: 39010100
26. Ahumada-Canale A, Jeet V, Bilgrami A, Seil E, Gu Y, Cutler H. Barriers and facilitators to implementing priority setting and resource allocation tools in hospital decisions: A systematic review. Soc Sci Med. 2023;322:115790. https://doi.org/10.1016/j.socscimed.2023.115790 PMID: 36913838
27. Shin MH, Montano A-RL, Adjognon OL, Harvey KLL, Solimeo SL, Sullivan JL. Identification of Implementation Strategies Using the CFIR-ERIC Matching Tool to Mitigate Barriers in a Primary Care Model for Older Veterans. Gerontologist. 2023;63(3):439–50. https://doi.org/10.1093/geront/gnac157 PMID: 36239054
28. Bonawitz K, Wetmore M, Heisler M, Dalton VK, Damschroder LJ, Forman J, et al. Champions in context: which attributes matter for change efforts in healthcare?. Implement Sci. 2020;15(1):62. https://doi.org/10.1186/s13012-020-01024-9 PMID: 32762726
29. Miech EJ, Rattray NA, Flanagan ME, Damschroder L, Schmid AA, Damush TM. Inside help: An integrative review of champions in healthcare-related implementation. SAGE Open Med. 2018;6:2050312118773261. https://doi.org/10.1177/2050312118773261 PMID: 29796266
30. Powell BJ, Waltz TJ, Chinman MJ, Damschroder LJ, Smith JL, Matthieu MM, et al. A refined compilation of implementation strategies: results from the Expert Recommendations for Implementing Change (ERIC) project. Implement Sci. 2015;10:21. https://doi.org/10.1186/s13012-015-0209-1 PMID: 25889199
31. Medves J, Godfrey C, Turner C, Paterson M, Harrison M, MacKenzie L, et al. Systematic review of practice guideline dissemination and implementation strategies for healthcare teams and team-based practice. Int J Evid Based Healthc. 2010;8(2):79–89. https://doi.org/10.1111/j.1744-1609.2010.00166.x PMID: 20923511
